# Safety of Post-transplant Cyclophosphamide as a Single Agent for Graft-Versus-Host Disease Prophylaxis After Human Leukocyte Antigen (HLA)-Matched Transplantation With the Japanese Population: A Single-Center Phase Ⅰ/Ⅱ Study

**DOI:** 10.7759/cureus.100251

**Published:** 2025-12-28

**Authors:** Miki Joyce, Naoki Kurita, Bryan J Mathis, Kenichi Makishima, Sakurako Suma, Yuya Sasaki, Yasuhito Suehara, Keiichiro Hattori, Tatsuhiro Sakamoto, Takayasu Kato, Naoshi Obara, Shigeru Chiba, Hidekazu Nishikii, Mamiko Sakata-Yanagimoto

**Affiliations:** 1 Department of Hematology, Graduate School of Comprehensive Human Sciences, University of Tsukuba, Tsukuba, JPN; 2 Department of Hematology, University of Tsukuba Hospital, Tsukuba, JPN; 3 Department of Hematology, Institute of Medicine, University of Tsukuba, Tsukuba, JPN; 4 Department of Cardiovascular Surgery, Institute of Medicine, University of Tsukuba, Tsukuba, JPN; 5 Division of Advanced Hemato-Oncology Transborder Medical Research Center, University of Tsukuba, Tsukuba, JPN

**Keywords:** bone marrow stem cell transplantation, calcineurin inhibitor, graft-versus-host disease (gvhd), hla-matched donor, post-transplant cyclophosphamide

## Abstract

Background

Graft-versus-host disease (GVHD) is a severe complication after allogeneic hematopoietic stem cell transplantation (allo-HSCT). Although calcineurin inhibitor (CNI)-containing GVHD prophylaxis is currently standard, it has the disadvantages of nephrotoxicity, a risk for thrombotic microangiopathy, drug interaction, and a requirement for monitoring blood concentration. Therefore, we conducted a prospective trial with the Japanese population undergoing allo-HSCT from an HLA-matched donor to investigate the safety and preliminary efficacy of single-agent post-transplant cyclophosphamide (PTCy) for GVHD prophylaxis.

Methods

This single-center phase I/II trial was a prospective study registered in the UMIN Clinical Trials Registry (UMINID: UMIN000028779) on November 1, 2017, and enrolled patients aged 16-60 years old with hematological malignancies in remission but without a history of previous allo-HSCT. Myeloablative conditioning (fludarabine 120 mg/m^2^ and total body irradiation 12 Gy) was followed by bone marrow transplantation from an HLA-matched donor. Cyclophosphamide (50 mg/kg) was administered on days 3 and 4. The primary endpoint was GVHD-free, relapse-free survival (GRFS) at six months after transplantation. Secondary endpoints included overall survival, incidences of relapse, non-relapse mortality (NRM), and acute and chronic GVHD (a/cGVHD). The prespecified sample size was 20.

Results

Four patients were enrolled, and one patient withdrew due to medication non-compliance. The GRFS at six months post-transplant was 2 out of 3 evaluable patients (66.7%). Grade 2 aGVHD was observed in 2/3 (66.7%), and neither grade 3-4 aGVHD nor moderate-to-severe cGVHD appeared within six months after transplantation. Two patients required additional cyclosporine administration because of hemophagocytic lymphohistiocytosis and grade 2 skin aGVHD, respectively, which improved promptly after cyclosporine administration. One patient relapsed, and NRM was not observed. Overall survival at six months was 3/3 (100%). The study was terminated early with poor recruitment.

Conclusions

PTCy alone may ensure safety, but PTCy alone may ensure safety. However, in this small cohort, PTCy monotherapy failed to provide adequate immunosuppression, necessitating CNI-based therapy in two of three patients for immune-mediated complications. Consistent with prior studies in HLA-matched settings that have not shown superiority of PTCy monotherapy over PTCy plus CNI, GVHD prophylaxis with single-agent PTCy should be carefully considered.

## Introduction

Allogeneic hematopoietic stem cell transplantation (allo-HSCT), albeit effective for refractory hematologic malignancies due to the graft-versus-leukemia (GVL) effect, carries a risk of graft-versus-host disease (GVHD). The incidence of grade 2 to 4 acute GVHD (aGVHD) after allo-HSCT is 40-50%, with 15% of patients developing grade 3 to 4 aGVHD regardless of prophylaxis [1]; this effect correlates with increased non-relapse mortality (NRM). Chronic GVHD (cGVHD) also occurs in approximately 30-70% of transplantees [2], causing impaired quality of life.

The current standard GVHD prophylaxis includes calcineurin inhibitors (CNI) [1] that act specifically on T cells, binding to intracellular calcineurin to suppress cytokine production and arrest the inflammatory cascade [3]. However, CNI requires frequent monitoring of blood concentration and has multiple drug interactions, plus risks for impaired kidney function, hypertension, calcineurin-inhibitor-induced pain syndrome, thrombotic microangiopathy, and posterior reversible encephalopathy syndrome [4]. According to a recent report, CNI also inhibits donor T cell exhaustion, leading to prolonged immune responses and an increased risk of developing chronic GVHD [5].

Recently, the efficacy of using post-transplant cyclophosphamide (PTCy) for GVHD prophylaxis was reported, particularly in human leukocyte antigen (HLA)-haploidentical transplants, which carry a high risk of GVHD [6]. Donor T cells, which are activated in response to recipient alloantigen, are highly sensitive to cyclophosphamide and selectively eliminated by PTCy [7]. PTCy alone was shown to be feasible in HLA-matched transplantation in certain conditions, in which no additional immunosuppressive drugs were needed in about 30-50% of the recipients [8, 9]. A higher incidence of GVHD was observed with reduced-intensity conditioning (RIC), or with peripheral blood stem cells (PBSC) as the graft when PTCy was used for GVHD prophylaxis alone in HLA-matched transplantation [7, 10].

In spite of differences between Japanese (who have a reduced risk of severe aGVHD [11]) and Caucasians, little is known about whether PTCy alone is sufficient for the Japanese population in an HLA-matched setting. Therefore, we conducted a prospective study that evaluated the safety and preliminary efficacy of PTCy monotherapy as GVHD prophylaxis in bone marrow transplantation (BMT) from HLA-matched donors with myeloablative conditioning in the Japanese population.

## Materials and methods

Study design and patients

This was a phase I/II, single-center study and was performed at the University of Tsukuba Hospital in Tsukuba, Japan. This study was conducted in accordance with the Declaration of Helsinki and recommendations for Interventional Trials (SPIRIT) 2013 Statement [12]. This prospective study was approved by the University of Tsukuba Hospital Institutional Review Board (CRB3180028) and registered in the Japan Registry of Clinical Trials (jRCTs031180140) and UMIN Clinical Trials Registry (UMIN000028779). Each participant received a Unique Patient Number (UPN) for identification.

The primary endpoint was GVHD-free, relapse-free survival (GRFS) at six months after BMT, defined as no deaths, relapse, development of grade 3 or higher aGVHD, or development of severe chronic GVHD by NIH criteria [13,14] within six months after transplantation. Secondary endpoints included overall survival, hematopoietic recoveries, incidences of grade 2 or higher aGVHD, mild-to-moderate cGVHD, relapse, and NRM. The Common Terminology Criteria for Adverse Events version 4.0 was used to assess treatment-related toxicity for the assessment of adverse events.

Patients aged 16-60 years and diagnosed with hematological malignancies in remission at the time of enrollment, who had no history of previous HSCT, presented with adequate organ function, and provided written, informed consent before enrollment were eligible for this study.

Conditioning, GVHD prophylaxis, and supportive care

BMT from an HLA-allele 8/8-matched related or unrelated donor followed a myeloablative conditioning consisting of 120 mg/m^2^ of fludarabine (30 mg/m^2^ intravenously on days -7 to -4) and 12 Gy of total body irradiation (TBI; 2 Gy twice per day on days -3 to -1) [15]. For GVHD prophylaxis, cyclophosphamide was administered at 50 mg/kg on days 3 and 4 after transplantation. To prevent hemorrhagic cystitis, adequate diuresis was performed, and mesna was used. Granulocyte-colony stimulating factor (5 mcg/kg/day) was given, starting on day 5, and continued until the absolute neutrophil count was greater than 0.5×10^9^/L for 3 consecutive days. Red blood cell and platelet transfusions were performed according to the Association for the Advancement of Blood and Biotherapies clinical practice guidelines [16,17]. All supportive care measures were performed in accordance with institutional practice and included routine anti-microbial prophylaxis against bacterial, fungal, *Pneumocystis jirovecii*, and herpes simplex infections.

Power testing

In an analysis using a Japanese transplant registry database, GRFS at six months after transplantation was approximately 60% [18]. Using this value as a reference, if we define the threshold GRFS as 30%, the expected GRFS as 60%, the alpha error as 0.05, and the beta error as 0.20, the number of eligible patients required for this clinical trial by a one-tailed test would be 17 patients. The number of patients required was deemed to be 20, based on the expectation that approximately 10% of the patients would typically be found ineligible for enrollment.

## Results

Patient characteristics

**Figure 1:**
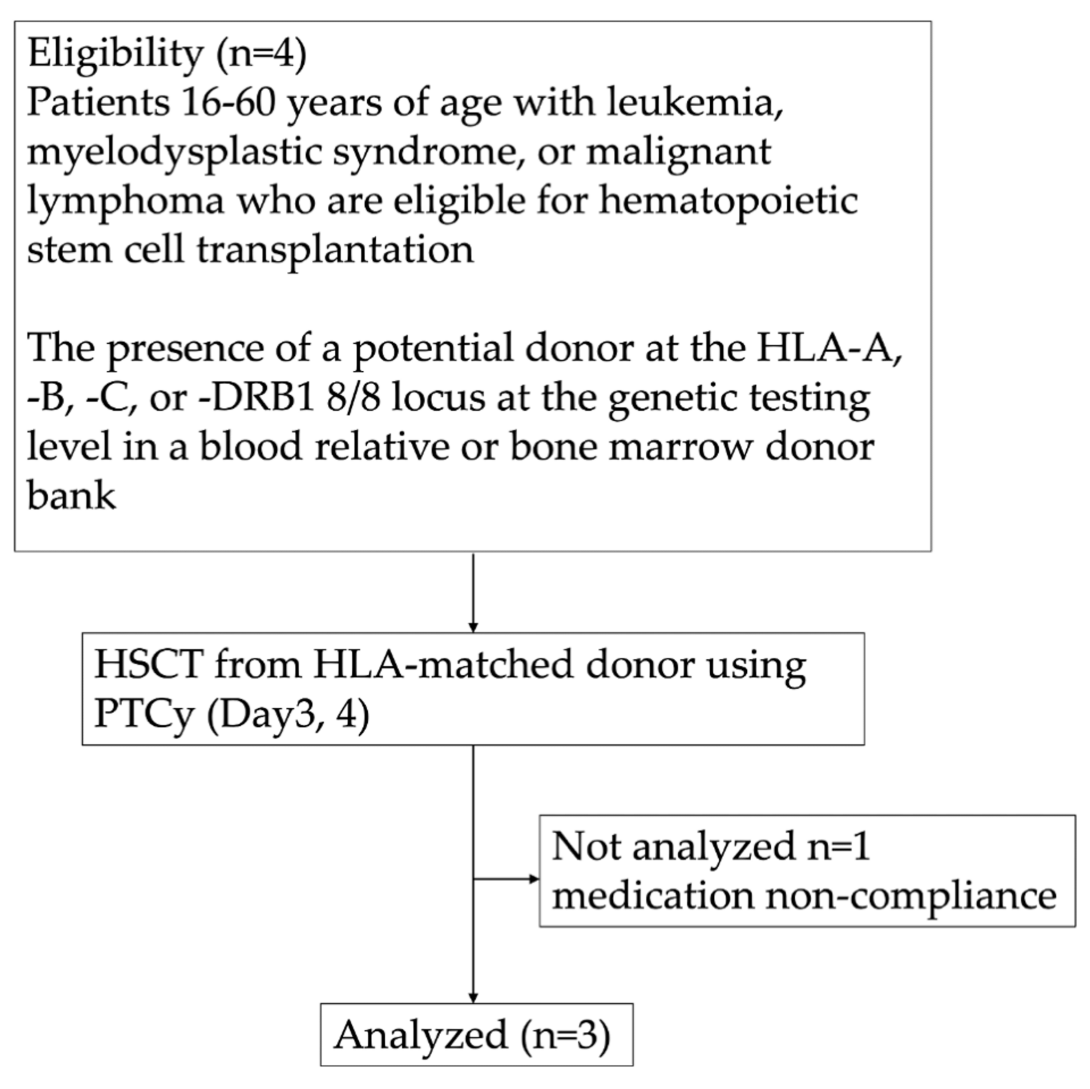
Study flow diagram Four patients were included and one out of four participants dropped out of the study during the observation period.

Four patients (UPN 1, 2, 3, and 4) were enrolled between November 2017 and May 2024 (Figure 1), with a median age of 40 years (range, 37-52). The diagnoses were acute myeloid leukemia (AML, n = 3) and acute lymphoblastic leukemia (n = 1) in remission. Of the four total patients, three received transplants from an HLA-matched related donor, and the fourth patient had an HLA-matched unrelated donor. All patients received myeloablative conditioning containing 12 Gy of TBI, followed by BMT, and PTCy as the single GVHD prophylaxis. A summary of the patient enrollment procedure is given in Figure 1, and the characteristics are summarized in Table 1. One patient out of the four quit on day 48 after BMT. This patient developed dissatisfaction related to prior-treatment neuropathy and post-transplant infections and repeatedly declined oral administration of required supportive care medications and communication with the medical staff. Liver dysfunction emerged from day 42, and hepatic GVHD was suspected, but the patient refused a liver biopsy. As additional immunosuppression would have been required if GVHD was confirmed, and this would violate the protocol, we could not ensure trial safety without diagnostic evaluation; therefore, the patient was withdrawn from the study. The study was terminated early due to poor recruitment in May 2024.

**Table 1:** Patient characteristics UPN, unique patient number; AML, acute myeloid leukemia; ALL, acute lymphoid leukemia; PS, performance status; CR, complete remission; RI, remission-induction therapy; C, consolidation therapy; MAC, myeloablative conditioning; Flu, fludarabine (30mg/m^2^); TBI, total body irradiation; NCC, nucleated cell count

UPN	1	2	3	4
Age, years	37	52	38	42
Sex	Male	Male	Male	Male
PS	0	0	0	0
Disease	AML (CR1)	AML (CR1)	ALL (CR1)	AML (CR2)
Previous treatment	RI 1, C 1-2	RI 1, C 1-3	RI 1, C 1-3	RI 1, C 1-4
				RI 1, C 1-3
Conditioning (MAC)	Flu+TBI 12 Gy	Flu+TBI 12 Gy	Flu+TBI 12 Gy	Flu+TBI 12 Gy
Donor	Male, related	Male, unrelated	Male, unrelated	Male, unrelated
ABO mismatch	match	match	match	Major mismatch
NCCs (×10^8^ cells/kg)	192	110	230	107

Primary and secondary endpoints

The GRFS in six months after BMT as the primary endpoint was 66.7% (two out of three evaluable cases) (Table 1), since one of the patients (UPN1) experienced relapse five months after transplantation. The engraftment rate was 100% and the median time to engraftment was 17 days (range, 15-28), including one patient (UPN4) who showed delayed engraftment on day 28 in the context of hemophagocytic lymphohistiocytosis (HLH), an immune-mediated manifestation of excessive immune dysregulation under PTCy alone that ultimately required additional CNI therapy. Grade 1 and 2 skin aGVHD were observed in 2 (UPN1 and 4) and 1 (UPN3) cases, respectively. No grade 3-4 aGVHD nor moderate-to-severe cGVHD occurred within 6 months after transplantation. Relapse and NRM were 1/3 (33.3%) and 0/3 (0%), respectively. Overall survival at 6 months was 3/3 (100%). The data on response and outcomes are summarized in Table 2.

**Table 2:** Response and outcome UPN, unique patient number;  n.d., not detected; N/A, not applicable; CsA, cyclosporine

UPN	1	2	3	4
Day of neutrophil ≥ 0.5 × 10^9^ /L	17	15	17	28
Day of platelet ≥ 20 × 10^9^ /L	33	26	36	35
Day of platelet ≥ 50 × 10^9^ /L	47	38	48	35
Relapse (time)	+ (5 months)	-	-	-
Follow-up period	39 months, alive	Withdrawn (day 48)	65 months, alive	38 months, alive
Death due to adverse events	N/A	N/A	N/A	N/A
Other immunosuppressant	N/A	N/A	CsA	CsA

Adverse events and discontinuations

**Figure 2:**
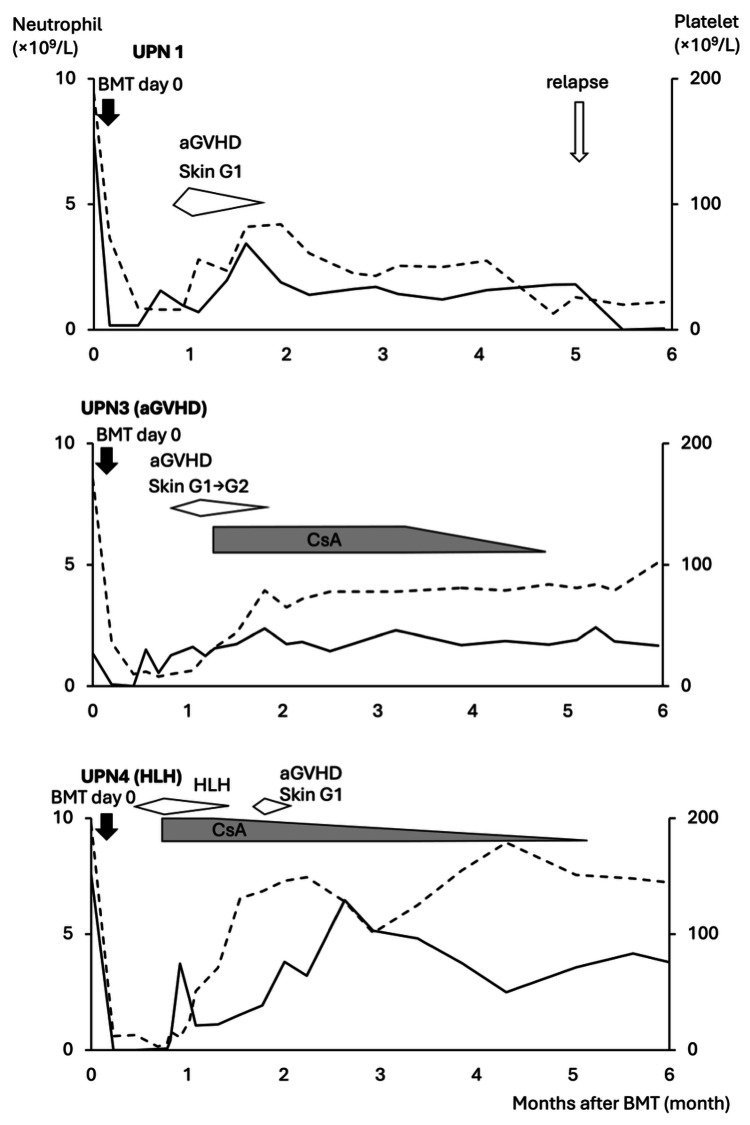
Clinical course and hematopoietic recovery of each patient until six months after bone marrow transplantation Solid and dotted lines indicate neutrophil (left axis) and platelet (right axis) levels, respectively. Arrows indicate post-transplant cyclophosphamide. aGVHD occurred in UPN 1 and 3. Hemophagocytic lymphohistiocytosis occurred in UPN 4. Cyclosporine administration was required as a treatment in UPN 3 and 4. UPN1 had a relapse within six months. UPN, unique patient numbers; aGVHD, acute GVHD Images created by the authors using MS PowerPoint (Microsoft Corp., USA)

Grade 3 febrile neutropenia occurred in all cases. Two patients required additional immunosuppressant (cyclosporine) because of aGVHD (UPN3) and HLH (UPN4). For UPN3, grade 2 skin aGVHD was observed on day 36, and cyclosporine was started on day 40 (Table 3). For UPN4, lactate dehydrogenase (LDH) and ferritin were elevated on day 24. With the delayed engraftment and unexplained fever, HLH was diagnosed by the massive macrophage infiltration and hemophagocytosis in the bone marrow. After cyclosporine was added on day 24, LDH decreased, and neutrophil engraftment was confirmed on day 28 (Table 4). Although grade 1 acute skin GVHD occurred temporally during the tapering of cyclosporine, it was successfully discontinued on day 171. The clinical trajectories of the three patients who did not withdraw are presented in Figure 2.

**Table 3:** Hematological/non-hematological toxicities aGVHD, acute graft-versus-host disease; cGVHD, chronic graft-versus-host disease

	Grade ≥ 3
aGVHD	0
cGVHD	0
Hemophagocytic lymphohistiocytosis	1
Febrile neutropenia	3
Anal pain	1

**Table 4:** Trends in LDH and ferritin in UPN4 In UPN4, LDH and ferritin rose on day 24; HLH was diagnosed and cyclosporine initiated that day, after which LDH declined and neutrophil engraftment occurred by day 28. LDH, lactate dehydrogenase; HLH, hemophagocytic lymphohistiocytosis

Day	21	24	28	31	40
LDH (U/L)	471	1362	862	602	369
Ferritin (ng/mL)	-	64100	25800	10960	3900

## Discussion

Here, we tested the safety of single immunosuppression via PTCy in Japanese allo-HSCT patients who received BMT from HLA-matched donors after myeloablative conditioning. However, the study did not meet recruitment goals for two reasons. First, our low patient enrollment was due to the influence of the COVID-19 pandemic. Second, several papers that demonstrated negative results on the superiority of PTCy alone were published during the study period [19, 20]. For these reasons, the trial was terminated after only four cases.

In our study, one patient did not require the use of other immunosuppressive agents, while the other two patients needed additional immunosuppressants for HLH and grade 2 skin acute GVHD, respectively. Reports indicate that the expected frequency of post-HSCT HLH is 3% [21]. Considering that HLH leading to death before neutrophil engraftment has been reported to occur frequently with weaker immunosuppression [22] and that the HLH and delayed neutrophil recovery in our case responded promptly to cyclosporine administration, the HLH we encountered may have been caused by inadequate immunosuppression with PTCy alone. Since the use of reduced-intensity conditioning or peripheral blood as a stem cell source with single PTCy has been reported to frequently facilitate aGVHD [23], we used a combination of myeloablative conditioning and BM. However, in spite of these measures, PTCy alone may be insufficient to prevent excessive immune reactions. In fact, in this study, HLH developed in 2 of the 3 patients. In this context, HLH should be regarded not merely as an adverse event but as a clinical manifestation of excessive immune dysregulation. Failure to eliminate antigens driving autoimmune and autoinflammatory processes can lead to inappropriate immune stimulation, triggering a cytokine storm and resulting in HLH [21]. These observations raise the possibility that single-agent PTCy is not sufficient as GVHD prophylaxis in patients with strong underlying immune activation. More intensive or combined regimens may therefore be required in such settings.　Recently, excellent outcomes from multiple-drug combinations, including CNIs and PTCy, were reported [24]. Moreover, combination regimens of PTCy plus 2 immunosuppressive drugs (with a single CNI) decreased the risk of severe cGVHD, reduced mortality, and improved overall survival [20]. Although CNI has potential risk for impaired kidney function, hypertension, CNI-induced pain syndrome, thrombotic microangiopathy, and posterior reversible encephalopathy syndrome [4], putative benefits from omitting CNI may come at the cost of increasing excessive and errant immune reactivity. 

In the effort to reduce the adverse effects of CNIs, several novel combination therapies are being developed in a different direction from PTCy alone. To reduce side effects of CNIs, sirolimus has been reported as a comparable alternative to tacrolimus in combination with PTCy and mycophenolate mofetil for GVHD prophylaxis in HLA-haploidentical peripheral blood stem cell transplantation [25]. It has also been tested for safety and effectiveness in combination with dipeptidyl peptidase-4 (DPP-4), with a recent report showing that DPP-4, plus tacrolimus and sirolimus, resulted in lower incidence of aGVHD after myeloablative allo-HSCT [26]. As DPP-4 is involved in a broad range of biological processes, including hematopoietic cytokine activity and T-cell immune function, it could serve as the basis of a CNI-free cocktail that attacks donor T-cell activation on multiple fronts to more effectively suppress GVHD without harsh CNI side effects.

Our trial must acknowledge limitations. First, the recruitment was low, resulting in an absence of statistical power. In spite of this, the low severity of adverse events (including mortality) seen in the enrolled patients may reassure physicians that PTCy could be a component of a CNI-free multidrug regimen that offers some benefits without unbalancing risk profiles.

## Conclusions

PTCy can be safely included in the pursuit of CNI-free, post-HSCT immunosuppressive regimens without severe GVHD or NRM, although, alone, it may not be sufficient to suppress excessive autoimmune reactions. We concluded that, in Japanese patients, PTCy may be able to ensure the safety of bone marrow transplantation with myeloablative conditioning from an HLA-matched donor when CNI is appropriately added without delay for immune-mediated complications such as HLH or GVHD.
